# Six-Week Supplementation with Creatine in Myalgic Encephalomyelitis/Chronic Fatigue Syndrome (ME/CFS): A Magnetic Resonance Spectroscopy Feasibility Study at 3 Tesla

**DOI:** 10.3390/nu16193308

**Published:** 2024-09-30

**Authors:** Beata R. Godlewska, Amy L. Sylvester, Uzay E. Emir, Ann L. Sharpley, William T. Clarke, Marieke A. G. Martens, Philip J. Cowen

**Affiliations:** 1 Department of Psychiatry, University of Oxford, Oxford OX3 7JX, UK; amy.sylvester@maastrichtuniversity.nl (A.L.S.); ann.sharpley@psych.ox.ac.uk (A.L.S.); marieke.martens@psych.ox.ac.uk (M.A.G.M.); phil.cowen@psych.ox.ac.uk (P.J.C.); 2 Oxford Health NHS Foundation Trust, Oxford OX4 4XN, UK; 3 Department of Psychiatry and Neuropsychology, Mental Health and Neuroscience Research Institute, Maastricht University, 6229 ER Maastricht, The Netherlands; 4 Wellcome Centre for Integrative Neuroimaging, University of Oxford, Oxford OX1 2JD, UK; uzay_emir@med.unc.edu (U.E.E.); william.clarke@ndcn.ox.ac.uk (W.T.C.); 5 Department of Radiology, University of North Carolina School of Medicine, Chapel Hill, NC 27599, USA

**Keywords:** myalgic encephalomyelitis/chronic fatigue syndrome (ME/CFS), creatine monohydrate, supplementation, MRS, cognition, hand grip strength

## Abstract

Background: Myalgic encephalomyelitis/chronic fatigue syndrome (ME/CFS) is a chronic medical condition with no specific pharmacological treatment. Creatine, a nutrient essential for maintaining energy homeostasis in the cells, is a candidate for interventions in ME/CFS. Methods: Fourteen participants with ME/CFS received supplementation with 16 g creatine monohydrate for 6 weeks. Before starting creatine and on the last day of treatment, participants underwent brain magnetic resonance spectroscopy (MRS) scanning of the pregenual anterior cingulate cortex (pgACC) and dorsolateral prefrontal cortex (DLPFC), followed by symptom, cognition, and hand-grip strength assessments. Results: Eleven participants completed the study. Creatine treatment increased creatine concentration in both the pgACC and DLPFC (*p* = 0.004 and 0.012, respectively), decreased fatigue and reaction time (RT) on congruent and incongruent trials of the Stroop test (*p* = 0.036 and 0.014, respectively), and increased hand-grip strength (*p* = 0.0004). There was a positive correlation between increases in pgACC creatine and changes in RT on Stroop congruent and incongruent trials (*p* = 0.048 and *p* = 0.022, respectively). Creatine was well tolerated, and none of the participants stopped treatment. Conclusion: Creatine supplementation over six weeks in ME/CFS patients increased brain creatine and improved fatigue and some aspects of cognition. Despite its methodological limitations, this study encourages placebo-controlled investigations of creatine treatment in ME/CFS.

## 1. Introduction

Myalgic encephalomyelitis/chronic fatigue syndrome (ME/CFS) is a chronic medical condition characterised by fatigue unrelated to exertion and not relieved by rest, post-exertional malaise (PEM), and cognitive dysfunction (often experienced as ‘brain fog’) [1]. ME/CFS, with a prevalence around 1% worldwide (exact estimates depending on participants’ characteristics, such as gender and age, case definitions, and diagnostic methods), is a relatively common disorder, approximately equivalent to major health disorders such as schizophrenia and heart failure and with a higher prevalence than dementia, epilepsy, or rheumatoid arthritis [2,3]. It is 1.5–2 times more prevalent among women than men [2]. Apart from its commonality, ME/CFS is associated with a high level of personal and societal burden [4,5]. Despite the above, there are no specific licensed pharmacological treatments, and research in this area is still scarce.

Creatine, as a nutrient essential for maintaining energy homeostasis in the cells across the body, including in the brain [6,7], is a promising candidate for interventions in ME/CFS [8,9,10]. The primary role of creatine is as an acceptor of high-energy phosphate in a reaction catalysed by creatine kinase that creates phosphocreatine (creatine + ATP <-> PCr + ADP + H^+^; ATP—adenosine triphosphate, ADP—adenosine diphosphate, PCr—phosphocreatine). Phosphocreatine acts as an energy buffer, quickly and easily diffusing to sites of demand to regenerate ATP [11]. As such, creatine availability is critical for energy-demanding tissues such as brain and muscle. Creatine also supports mitochondrial health due to its antioxidant and neuroprotective properties, and dysfunction in both has been suggested in ME/CFS [12,13,14]. A recent 7 tesla (7T) magnetic resonance spectroscopy (MRS) study showed lower creatine concentrations in the pregenual anterior cingulate cortex (pgACC) of individuals with ME/CFS compared to healthy controls [15].

To the best of our knowledge, there are no published studies on how creatine supplementation affects creatine levels in the brain in ME/CFS. Of potential relevance to ME/CFS may be a small trial in long COVID, a post-viral syndrome also characterised by persistent fatigue and brain-fog, which showed a significant reduction in fatigue, and an increase in creatine levels at the frontal and parietal white matter after 3 months (respectively, 5.1 vs. −1.7% on the placebo and 30.2% vs. −2% on the placebo) and 6 months (respectively, no further change on creatine and the placebo and −1.4% vs. +2% on the placebo) of supplementation with 4 g of creatine monohydrate per day [16].

This study was designed to assess the feasibility of a six-week intervention of 16 g daily creatine monohydrate supplementation in people with ME/CFS. The primary aim was to assess the effect of oral creatine dietary supplementation on brain creatine levels in ME/CFS patients using MRS. Secondary aims included an assessment of the relationship between changes in the brain creatine concentration over treatment and changes in clinical symptoms, cognitive performance, and hand-grip strength.

## 2. Materials and Methods

### 2.1. Intervention and Justification for the Dose/Intervention Duration

Oral supplementation with 16 g creatine monohydrate daily for six weeks was applied in the study. As there are no studies in ME/CFS populations suggesting which dose would lead to an increase in the brain creatine concentration, we based the choice of the dose and the length of treatment on studies in healthy and depressed individuals [17]. They showed that doses in the range of 2–20 g/day applied for 5 days to 8 weeks led to a 3–10% increase in the brain creatine levels. 

The following three factors were also taken into account when deciding the dose and duration of the intervention in the current study: (1) higher doses (10–20 g/day) and longer supplementation periods seemed to consistently produce increases in the brain creatine level in other populations (the only MRS studies that showed no increase in creatine were those using lower doses) [9]; (2) it was suggested that a dose and treatment period may need to be higher to influence disease states, in particular creatine deficiencies and mitochondrial-related diseases, and mitochondrial dysfunction was suggested in ME/CFS [18]; and (3) it is possible that ME/CFS might be associated with blood–brain barrier dysfunction [19].

### 2.2. Participants and Clinical Ratings

The study obtained ethical approval from the National Research Ethics Service Committee (NRES), South-Central Oxford. In total, there were 14 participants with ME/CFS (10 females, 4 males, mean age 40.6 years, range 19–61 years). All participants gave full informed written consent. The study involved two visits, between which the participants took orally 16 g creatine monohydrate daily for 6 weeks. The second visit fell on the last day of supplementation. At each visit, participants underwent an MRS scan, a computerised cognitive assessment, a hand-grip strength measurement, and an assessment of symptoms using questionnaires.

Participants were recruited from the local community through newspapers and social media advertisements, word of mouth, and among participants in previous studies between November 2019 and August 2022. Four participants were recruited before COVID-19-related restrictions were introduced: two completed the study, and two had only the first visit due to research being suspended. The remaining ten participants were recruited between February and August 2022. In all participants, the diagnosis of ME/CFS had been made originally by an appropriate professional (ME/CFS service); additionally, a clinically trained member of the research team (B.R.G.) confirmed the presence of the symptoms at the time of the study according to the Center for Disease Control and Prevention 94 (CDC-94) criteria for ME/CFS [20]. This included the requirement of symptoms lasting at least 6 months and the definite onset of symptoms (i.e., not lifelong). The full list of CDC diagnostic criteria for ME/CFS used in this study is available in Supplementary Table S2. Exclusion criteria included current psychiatric comorbidities as diagnosed by DSM-5 [21] (for example, current major depression, bipolar disorder, anxiety disorder, psychotic disorder, eating disorder), any general medical conditions known to influence energy processing and/or linked to fatigue, current creatine dietary supplementation, current treatment with any medication/supplementation likely to interfere with energy metabolism, known kidney and/or liver problems, contra-indications to magnetic resonance imaging (MRI), and pregnancy or breast feeding. The current and past mental health history was determined by a structured interview (the Structured Clinical Interview for DSM-5, SCID-5) [21].

Fatigue was measured with the Chalder Fatigue Scale (ChFS) [22] and Fatigue Severity Scale (FSS) [23], and mood was measured with the Beck Depression Inventory II (BDI-II) [24]. We also assessed two other key ME/CFS symptoms: sleep using Pittsburgh Sleep Quality Inventory (PSQI) [25] and pain with Brief Pain Inventory (BPI; severity and interference scores) [26].

### 2.3. Magnetic Resonance Spectroscopy

**Figure 1:**
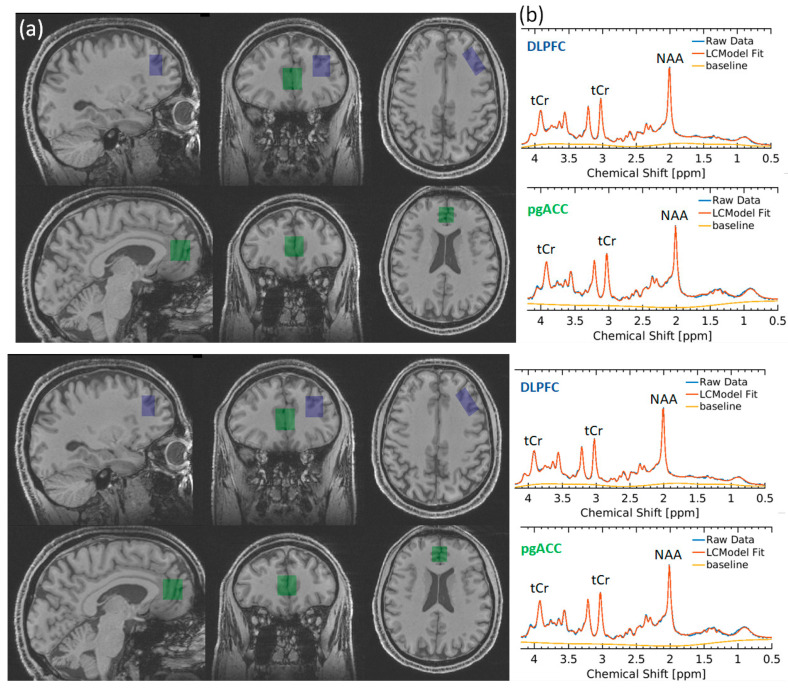
(**a**) Voxel placement in DLPFC (blue) and pgACC (green). (**b**) Representative spectra from the DLPFC (blue) and pgACC (green). tCr total creatine, NAA N-acetylaspartate.

Participants underwent proton (1H) MRS scanning at the Wellcome Centre for Integrative Neuroimaging in Oxford. Scanning was performed on a 3 T Siemens Prisma scanner (Siemens Healthineers, Erlangen, Germany) equipped with a Siemens Healthineers 32 channel head coil. Spectra were measured from a voxel in the pregenual anterior cingulate cortex (pgACC, 20 × 20 × 20 mm) and dorsolateral prefrontal cortex (dlPFC 15 × 20 × 30 mm) (Figure 1). The voxel was positioned manually by reference to 1 mm isotropic T1-weighted MPRAGE image. First- and second-order shims were adjusted by gradient-echo shimming [27]. Spectra were measured by semi-adiabatic localisation using an adiabatic selective refocusing (semi-LASER) sequence (TE = 28 ms; TR = 4 s; 64 averages) with variable power radiofrequency pulses with optimised relaxation delay (VAPOR) [28] water suppression and outer volume saturation [29,30]. Unsuppressed water spectra acquired from the same voxel were used to remove residual eddy current effects and to combine the spectra from individual array coils. 

Neurometabolite concentrations were quantified with LCModel [31], with prior reported chemical shifts and coupling constants [32,33] as a basis for the model spectra of glutamine, glutamate, and GSH by using GAMMA/PyGAMMA simulation library of VESPA for applying the density matrix formalism (Versatile Simulation, Pulses, and Analysis 9). Fully resolved RF pulses and sequence timing were used to perform spatially resolved simulations. The model included a macromolecule spectrum acquired from the occipital cortex using an inversion recovery sequence (TR  =  2.5 s, TE  =  28 ms, inversion time TI  =  750 ms). The concentration was reported in molality units (μmol/g) and was estimated as a ratio to an unsuppressed water spectrum obtained from the same voxels [34]. The spectra quality requirements were CRLB ≤ 30% and full-width half-maximum height linewidth less than 2.5 × SD of the group mean. The MP-RAGE images were segmented using FSL FAST, and FSL-MRS was used to determine grey matter (GM), white matter (WM), and cerebrospinal fluid (CSF) fraction (fGM, fWM, fCSF) in the voxels [35]. The results of segmentations were visually inspected. Concentrations were then corrected for these with the following formula:

MetCorr = (MetConcAbs × (fGM × 43,300 + fWM × 35,880 + fCSF × 55,556))/1 − fCFS),

where MetCorr is the corrected concentration of a metabolite; MetConcAbs is the metabolite concentration from the uncorrected LCModel output; and fGM, fWM, and fCSF stand for, respectively, fractions of GM, WM, and CSF in the voxel of interest. Tissue volume fraction was performed for GM, WM, and CSF content, rather than CSF only, following reports of differences in creatine concentration in GM and WM [36].

### 2.4. Cognitive Function

Cognitive function was evaluated with the Stroop Colour and Word Test (called simply the Stroop test), N-back test, and Rey Auditory–Verbal Learning Test (RAVLT). The Stroop test assesses the ability to inhibit cognitive interference occurring when the processing of one stimulus feature interferes with the simultaneous processing of another feature of this stimulus [37]. In this study, participants were shown words ‘red’, ‘blue’, or ‘green’ on the computer screen; each of these words could be written in one of these colours (red, blue, or green). There were two types of trials: where the word name and font colour were the same and incongruent trials, where the word name and font colour were different. Participants were asked to press a button corresponding to the colour the word was written in (i.e., not what the word said). For example, if the word said red but was written in green ink, the participant was supposed to press the green button. Reaction time (RT) and accuracy were calculated. The calculated difference in reaction time between incongruent and congruent trials served as a measure of inhibitory control. The N-back task is considered to be a measure of working memory [38]. In the study, participants were presented with a sequence of letters appearing one after another on the screen (one letter at the time) and were asked to decide whether a letter currently on the screen matched the one that appeared n letters earlier: 1 letter earlier in the ‘1-back’ condition, 2 letters earlier in the ‘2-back’ condition, and 3 letters earlier in the ‘3-back’ condition. The RAVLT evaluates short-term memory, working memory, and long-term memory [39]. The RAVLT involved five sequential presentations of a 15-word list (List A, Trials 1 through 5). Each word was presented at the rate of one word per second. After each presentation, participants were asked to say the words they remembered (free recall list A). Subsequently, participants were presented with a new list of 15 words (list B) and asked to say the words they remembered from this list only (free recall list B). After that, they were asked to say the words they remembered from list A (short-delay free recall) and, after 15 min, to say all the words they remembered from list A again (long-delay free recall). Number of correctly recalled items was recorded for list A immediate free recall (each trial and the sum from all trials), short-delay free recall and long-delay free recall, list B immediate free recall, free recall intrusions (immediate and delayed), total repetitions (across all recall trials), repetition hits, and repetition false positives. 

### 2.5. Hand-Grip Strength Measurement

Hand-grip strength was measured using Jamar Hydraulic Hand Dynanometer by Patterson Medical (https://www.performancehealth.co.uk/jamar-hydraulic-hand-dynamometer, accessed on 20 September 2024). As per manufacturer’s instructions, participants were asked to sit with their shoulders adducted and neutrally rotated, elbow flexed at 90 degrees, forearm in neutral position, and wrist between 0 and 30 degrees dorsiflexion and between 0 and 15 degrees ulnar deviation. They were asked to squeeze the handle as hard as they could and then rest for 60 s. The scores of three successive trials were recorded, and the average was taken.

### 2.6. Statistics

Statistical analyses were performed in SPSS version 27. Normality was tested with the Shapiro–Wilk test. Differences before and after creatine supplementation were examined using paired-samples t-tests, and Wilcoxon Signed-Rank Test where distribution was not normal (the name of the test is indicated in the results section and tables). Effect size was estimated by Cohen’s d. As this was an exploratory feasibility study, there was no correction for multiple testing. Correlations between the change in creatine concentration and change in individual ratings of fatigue, clinical scores, and cognitive measures were carried out using Pearson’s product moment and were not corrected for multiple comparisons.

## 3. Results

### 3.1. Demographics and Clinical Data

Eleven participants (seven females and four males) out of fourteen completed the study. In two cases, participation was halted due to restrictions related to the COVID-19 pandemic. Another participant decided not to come for their second session due to travel distance. The mean age was 40.2 years (SEM 3.4, range 19.3–59.9 years). The mean weight was 73.6 kg (SEM 3.3, range 56–95 kg), mean height 172 cm (SEM 1.9, range 163–182 cm), and mean BMI 24.9 (SEM 1.1, range 19.6–30.9). All participants were non-smokers. At the first visit, BDI-II was 12.8 (range 5–38). However, none of the patients met the criteria for major depression at the clinical assessment, and items related to mental and physical fatigue accounted for higher scores on BDI-II in all but one case. Regarding prescription medications, one participant was taking candesartan (for hypertension), another salbutamol PRN (for asthma), and another was on hormone replacement therapy (oestrogen and progestogen). Other participants did not take prescription medications, and, apart from ME/CFS, had no other diagnoses.

Over the treatment period, there was a significant decrease in both ChFS and FSS scores (respectively, *p* = 0.031 and *p* = 0.038). Depression (BDI II), pain (BPI), and sleep (JSQI) scores numerically decreased, but changes were statistically non-significant (*p* > 0.05). See Table 1 for details. 

### 3.2. MRS 

One pgACC dataset was rejected due to poor quality. Therefore, the final analysis included pgACC data from 10 participants and DLPFC data from 11 participants. Example spectra are shown in Figure 1. For both the pgACC and DLPFC, there were no significant differences between the pre- and post-treatment signal-to-noise ratio (SNR), full-width half-maximum (FWHM), and GM, WM, and CSF content (all *p* > 0.5) (Table 1). However, in one participant in the pgACC and in another in the DLPFC, the FWHM difference between scans exceeded 0.024 ppm (this was considered in the analysis, see below).

After six weeks of creatine treatment, there was a statistically significant increase in total creatine concentration in both the pgACC (by 8.3%) and DLPFC (by 2.9%) (pgACC: 1st scan—mean 8.95 μmol/g, SEM 0.29; 2nd scan—mean 9.76 μmol/g, SEM 0.24; t(df 9) −3.844, *p* = 0.004; DLPFC: 1st scan—mean 6.94 μmol/g, SEM 0.10; 2nd scan—mean 7.15 μmol/g, SEM 0.28; t(df 10) −3.079, *p* = 0.012). The results remained significant after excluding data where the difference in FWHM between the scans exceeded 0.024 (pgACC: *p* = 0.01; DLPFC: *p* = 0.007). To check for potential changes in all metabolites, rather than specific to creatine, we analysed the difference in NAA levels, which was not significant (pgACC: *p* = 0.415; DLPFC: *p* = 0.215). See Table 2 for details.

### 3.3. Cognitive Scores

Due to technical problems, cognitive data were not available for all participants. The Stroop test data were available for nine participants and RAVLT data for eight participants.

After six weeks of treatment, we observed a significant reduction in reaction time (RT) on both congruent and incongruent trials of the Stroop test (respectively, t(df 8) = 2.524, *p* = 0.036, and t(df8) = 3.142, *p* = 0.014), and RT on 1-back trials on N-back task (t(8) = 2.650, *p* = 0.029). However, there was no statistically significant change in a measure of inhibitory control (RT on congruent the Stroop trial—RT on the incongruent Stroop trials) (t(df8) = 1.364, *p* = 0.210). There were no statistically significant changes (*p* > 0.05) in Stroop accuracy for the congruent and incongruent trials; N-back RT on 0-, 2-, and 3-back trials; and accuracy on all trials or any of AVLT parameters (correctly recalled items on immediate free recall, list B free recall, correctly recalled items on both short-delay and long-delay free recall, immediate and delayed free recall intrusions, total repetitions, long-delay recognition hits, and false positives). The results for all cognitive tests are presented in Supplementary Table S1.

### 3.4. Hand-Grip Strength

There was a significant increase in hand-grip strength (visit 1: mean 48.9, SEM 4.5; visit 2: mean 61.9, SEM 4.9; t(df6) = −7.201, *p* = 0.0004; effect size 2.722) following creatine treatment. 

### 3.5. Brain Metabolites and Demographics/Clinical Measures/Cognitive Scores 

There was a significant correlation between the change in pgACC creatine concentration and change in RT on both congruent (r = −0.670, *p* = 0.048) and incongruent (r = −0.742, *p* = 0.022) trials on the Stroop task as well as DLPFC creatine 3-back trials accuracy on N-back task (r = 0.776, *p* = 0.014). There were no other significant correlations for the change in creatine concentration, including with other cognitive function measures, clinical scores, and hand-grip strengths. 

### 3.6. Side Effects

Creatine was generally well tolerated, and none of the participants stopped treatment. One participant, however, gained weight during the study (5.2 kg), one reported nausea and vomiting, and one had problems sleeping if creatine was taken at night (this resolved when they took creatine earlier in the day).

## 4. Discussion

As far as we are aware, this is the first published investigation of the effect of creatine supplementation on brain creatine concentration in ME/CFS (a study protocol was recorded for another study, but the results have not been published; clinicaltrials.gov ID NCT02374112). As hypothesised, a six-week supplementation with 16 g creatine monohydrate daily significantly increased creatine concentration in the brain (by 8.3%, with an effect size of 1.215, in the pgACC and by 2.9%, with an effect size of 0.928, in the DLPFC). We also observed significant improvement in fatigue and hand-grip strength. There was a significant correlation between the change in pgACC creatine concentration and change in reaction time on both congruent and incongruent trials on the Stroop test and between the change in DLPFC creatine concentration and change in 3-back trial accuracy on the N-back task. However, there were no other significant correlations for the change in creatine levels, including with clinical scores, other cognitive function measures, and hand-grip strength.

There are reports of an increase in creatine concentration in the brain following oral supplementation with creatine monohydrate in healthy and depressed individuals [9] as well as in long COVID [16]. However, given reported blood–brain barrier (BBB) dysfunction in ME/CFS [19], a demonstration of the effectiveness of creatine transport from the blood to the brain is a relevant finding, especially in the context of future investigations. 

We chose two brain regions for our study, the pregenual ACC (pgACC, the rostral portion of the ACC) and DLPFC. The choice of the pgACC was based on the findings from our previous study [15], which showed decreased creatine concentration in this region in ME/CFS patients relative to heathy controls. The ACC has further been implicated in the pathophysiology of ME/CFS [40], and functional neuroimaging studies have shown ACC dysfunction in ME/CFS [41]. The ACC is crucial for associating information from different levels, including cognition, emotions, bodily sensations, and pain, and thus of high relevance to ME/CFS symptoms [42]. A recently proposed ‘neurocognitive framework’ identified the ACC as the key structure in the neural circuitry that weights up the costs and benefits of continued exertion in both cognitive and physical tasks [43]. This framework focused on the dorsal ACC; however, other studies suggested a similar importance of the pgACC for the assessment of the value of effort in decision-making [44]. The DLPFC was chosen because of its role in executive functioning and working memory. Cognitive problems, including memory, are among the most common complaints in ME/CFS, and DLPFC dysfunction in ME/CFS was shown in fMRI studies [38].

We showed that creatine concentration increased in both the pgACC and DLPFC after dietary supplementation, suggesting that an increase in creatine might occur across a number of the brain structures. However, as we only tested two regions, this hypothesis will need further testing. It should be noted that the Slankamenac et al. [16] study on long COVID showed an increase in creatine in only two out of thirteen tested brain regions and in white matter rather than grey matter. It is, however, not known how similar dysfunction in the BBB in long COVID and ME/CFS is despite the overlap of symptoms related to fatigue and PEM, and this may affect transport across the BBB. The MRSI study on ME/CFS by Mueller et al. [45] showed decreased creatine in only 1 out of 47 regions tested, the parietal cortex; this study, however, only compared people with ME/CFS and heathy controls and did not involve any interventions.

Although this is the only study on ME/CFS showing a change in brain creatine concentration in response to dietary supplementation with creatine monohydrate, it can be placed in the context of a few studies using creatine or creatine-related compounds in conditions sharing similarities with ME/CFS. For example, the above-mentioned long COVID trial [16] involving twelve patients (six on 4 g of creatine monohydrate per day and six on a placebo over six months) showed an increase in creatine in the skeletal muscle (vastus medialis muscle) and the brain (left frontal white matter and right parietal white matter) at 3 and 6 months. Creatine significantly decreased subjective feelings of fatigue and improved concentration; no correlations with changes in the creatine brain levels were presented. In a placebo-controlled 16-week trial using 20 g of creatine monohydrate for the first 5 days followed by 5 g per day in patients with fibromyalgia [46], creatine increased muscle phosphocreatine content and improved lower- and upper-body muscle strength, but no change in cognitive function, including Stroop test measures, was noted. A randomised controlled trial in women with CFS showed that 12-week supplementation with the creatine precursor guanidinoacetic acid increased muscle creatine concentration, functional performance, and specific fatigue subdomains [47]. One study [48] in patients with CFS failed to show benefits of a creatine-containing polynutrient supplement on fatigue severity and functional impairment. However, the dose used in this study was low (13 μg/100 mL, 125 mL single dose). 

In our study, participants reported an improvement in fatigue, reflected by a significant decrease in scores on self-reported fatigue severity and Chalder Chronic Fatigue Scales. This change, however, did not correlate with changes in creatine levels. With the lack of a placebo, it cannot be excluded that this subjective improvement was related to the study participation or taking a new treatment rather than to a specific benefit of creatine. This improvement is, however, consistent with the above-mentioned placebo-controlled trials [16,47]. Unfortunately, the only study that measured creatine change in the brain [16] in long COVID participants did not provide information on correlations between changes in fatigue and creatine levels.

Cognitive dysfunction (often reported as ‘brain fog’) [49] is among the most common and burdensome symptoms in ME/CFS [50]. Deficits regarding attention, memory, and reaction times were identified in meta-analyses of neuropsychological tests [50,51]. Creatine supplementation with creatine monohydrate was previously shown to have beneficial effects in a number of scenarios in which cognitive processes are stressed, for example, during sleep deprivation, experimental hypoxia, or the performance of more complex and thus more cognitively demanding tasks [17]. Functional MRI neuroimaging studies implicated both regions scanned, the ACC and DLPFC, in cognitive processing in ME/CFS, showing aberrant activation during the N-back task performance [52]. In our study, we observed a significant change over treatment in reaction time on both congruent and incongruent trials of the Stroop test, which correlated with the change in creatine concentration in the ACC. While the practice effect cannot be excluded, this correlation is intriguing in the context of ACC activation by the Stroop test shown by fMRI studies [53]. There was, however, no difference in inhibitory controlled measures as reaction time difference between incongruent and congruent trials on the Stroop task. A correlation was also noted between changes in accuracy on 3-back trials on the N-back task and creatine change in the DLPFC, which is involved in N-back performance; however, the change in N-back accuracy over treatment was non-significant. This is somewhat similar to the findings of fMRI studies, which showed that while DLPFC activity on demanding 2- and 3-back trials was lower in people with ME/CFS than healthy individuals, their actual performance was similar [52]. We did not observe any other significant changes in cognitive function measures apart from a possibly incidental improvement in reaction time on 1-back trials on the N-back test, which did not correlate with change in creatine and may be related to practice. Our results suggest that creatine has a limited impact on cognition in ME/CFS. However, it needs to be considered that either the duration of treatment or the dosage might have been inadequate. For example, in the Slankamenac et al. [16] study on long COVID, the protective effect of creatine against motivation loss was only seen after 6 months, but not at 3 months, of treatment. 

Supplementation with creatine monohydrate was associated with a highly significant increase in hand-grip strength. Although creatine is commonly used in sports to improve muscle function, to our knowledge, this is the first report of its ergogenic effect in ME/CFS [6]. We did not assess creatine concentration in the muscle; hence, we cannot directly relate the increase in hand-grip strength to its change. However, with some exceptions, studies in healthy individuals as well as the above-mentioned study on long COVID [16] consistently showed that supplementation with creatine monohydrate increased muscle creatine and phosphocreatine content by around 20% [6]. 

Despite the demonstrated feasibility and potential utility of creatine supplementation, this study was limited by the small number of patients, which may lead to false positives, especially as we made no correction for multiple comparisons given the exploratory nature of this study. Another limitation is the lack of a placebo, which does not allow for differentiation between the effect of creatine and the impact of other factors, such as participation in the trial. Also, given the lack of studies on the effect of creatine supplementation on the brain in ME/CFS and low number of such studies in other populations, we cannot be sure whether the dose and duration of supplementation chosen provides a potential effect in this study group. Finally, the detailed data on nutrition and physical training in participants are not available. However, participants reported that these did not change in any significant way during the study. 

## 5. Conclusions

To summarise, this study showed that creatine supplementation with a high dose of creatine over a medium-term period is feasible in ME/CFS and that it leads to an increase in creatine in the brain. Additionally, whilst it is suggested that creatine may have some effect on fatigue and cognition, these results need to be viewed with caution in light of the study limitations. However, this study encourages further investigations of creatine in ME/CFS, ideally using different doses and treatment durations with a placebo as a control.

## Figures and Tables

**Table 1:** Comparisons of clinical scores between visit 1 and visit 2 using paired-samples t-test. Mean (SEM). FSS—fatigue severity scale; ChFS—Chalder Fatigue Scale; BDI-II—Beck Depression Inventory II; PSQI—Pittsburgh Sleep Quality Inventory; BPI—Brief Pain Inventory. Effect size Cohen’s d is provided for statistically significant results.

Clinical Scores	Visit	Score	Paired-Samples *t*-Test Value	Paired-Samples *p*-Value	Effect Size Cohen’s d
FSS	1	53.64 (2.19)	2.395	0.038	0.722
	2	49.82 (3.19)			
ChFS	1	22.91 (1.33)	2.514	0.031	0.758
	2	16.55 (1.95)			
BDI-II	1	12.82 (2.69)	1.950	0.080	
	2	11.00 (3.18)			
PSQI	1	8.73 (0.49)	1.982	0.076	
	2	7.73 (0.69)			
BPI pain severity	1	2.75 (0.69)	1.049	0.335	
	2	2.07 (0.76)			
BPI pain interference	1	2.06 (0.61)	1.184	0.281	
	2	1.29 (0.59)			

**Table 2:** Comparisons between visit 1 and visit 2 using paired-samples t-test. Mean (SEM) absolute concentrations (μmol/g) of brain neurochemicals corrected for grey matter (GM), white matter (WM), and cerebrospinal fluid (CSF); GM, WM, and CSF content; spectra quality measures: full-width at half-maximum (FWHM) and signal-to-noise ratio (SNR). Significant results are in bold. Effect size Cohen’s d is provided for statistically significant results.

	Visit	Value	Paired-Samples *t*-Test Value	Paired-Samples *p*-Value	Effect Size Cohen’s d
Metabolite concentrations (μmol/g)					
ACC creatine	1	8.95 (0.29)	−3.844	**0.004**	1.215
	2	9.76 (0.24)			
DLPFC creatine	1	6.94 (0.10)	−3.079	**0.012**	0.928
	2	7.15 (0.28)			
ACC NAA	1	11.79 (0.32)	0.854	0.415	
	2	11.52 (0.24)			
PFC NAA	1	10.31 (0.10)	1.324	0.215	
	2	10.18 (0.10)			
Voxel content (fraction)					
ACC GM	1	0.5378 (0.0082)	0.280	0.560	
	2	0.5511 (0.0057)			
ACC WM	1	0.1258 (0.0061)	−0.324	0.753	
	2	0.1272 (0.0065)			
ACC CSF	1	0.3264 (0.0105)	0.760	0.467	
	2	0.3217 (0.0087)			
DLPFC GM	1	0.3663 (0.0135)	0.470	0.649	
	2	0.3625 (0.0122)			
DLPFC WM	1	0.5570 (0.0178)	−0.383	0.710	
	2	0.5613 (0.0160)			
DLPFC CSF	1	0.0767 (0.0078)	0.133	0.896	
	2	0.0763 (0.0230)			
Spectra quality measures					
ACC FWHM	1	0.050	0.000	1.000	
	2	0.050			
DLPFC FWHM	1	0.036 (0.002)	0.053	0.959	
	2	0.036 (0.004)			
ACC SNR	1	37.90 (1.85)	−0.614	0.554	
	2	39.6 (3.78)			
PFC SNR	1	61.64 (1.94)	0.525	0.611	
	2	59.55 (2.91)			

## Data Availability

The raw data supporting the conclusions of this article will be made available by the authors on request.

## Supplementary Materials

The following supporting information can be downloaded at https://www.mdpi.com/article/10.3390/nu16193308/s1, Table S1: Comparisons of cognitive measures between visit 1 and visit 2 using paired-samples t-test. Table S2. CDC-94 criteria for ME/CFS [20].
