# Supplementary material for: Six-Week Supplementation with Creatine in Myalgic Encephalomyelitis/Chronic Fatigue Syndrome (ME/CFS): A Magnetic Resonance Spectroscopy Feasibility Study at 3 Tesla
Source: Nutrients. 2024 Sep 30;16(19):3308. doi: 10.3390/nu16193308 (PMC11478479; doi:10.3390/nu16193308)
Supplement: Supplementary file 1 [file nutrients-16-03308-s001.zip › nutrients-3167607-supplementary.pdf]

Table S1. Comparisons of cognitive measures between visit 1 and visit 2 using paired-samples t-test. Mean (SEM). RAVLT – Rey Auditory-Verbal Learning Test. Effect size Cohen's d is provided for statistically significant results. NB the Stroop Test and N-back data were available for 9 participants, RAVLT data were available for 8 participants.

Psychological test	Visit	Score	Paired-samples t-test value	Paired-samples p value	Effect size Cohen's d
**Stroop Test**					
RT congruent trials	1	627.37 (23.09)	2.524	0.036	0.841
	2	536.32 (9.14)			
RT incongruent trials	1	708.71 (23.76)	3.142	0.014	1.047
	2	630.42 (15.94)			
Accuracy congruent trials	1	0.9900 (0.0037)	0.610	0.559	
	2	0.9878 (0.0028)			
Accuracy incongruent trials	1	0.9667 (0.0128)	-0.392	0.705	
	2	0.9700 (0.007)			
Inhibitory control (RT incongruent trials – RT congruent trials)	1	81.34 (21.99)	1.364	0.210	
	2	67.10 (14.03)			
**N-back**					
0-back RT	1	505.72 (21.02)	0.860	0.415	
	2	490.23 (13.10)			
1-back RT	1	608.94 (25.37)	2.650	0.029	0.883
	2	566.68 (18.55)			
2-back RT	1	648.26 (27.64)	1.799	0.110	
	2	617.76 (35.28)			
3-back RT	1	626.59 (31.22)	1.398	0.200	
	2	601.13 (27.94)			
0-back Accuracy	1	0.9389 (0.0207)	-1.423	0.193	
	2	0.9533 (0.0143)			
1-back Accuracy	1	0.9022 (0.0213)	0.293	0.777	
	2	0.8967 (0.0139)			
2-back Accuracy	1	0.7989 (0.0311)	-0.429	0.679	
	2	0.8078 (0.0237)			
3-back Accuracy	1	0.7967 (0.0401)	-0.232	0.823	
	2	0.8044 (0.0247)			

**RAVLT**				
Free Recall Correct				
Trial 1	1	7.00 (0.60)	0.000	1.000
	2	7.00 (0.63)		
Trial 2	1	10.50 (0.94)	0.704	0.504
	2	10.13 (0.58)		
Trial 3	1	11.88 (0.95)	0.000	1.000
	2	11.88 (0.74)		
Trial 4	1	13.00 (0.78)	-0.284	0.785
	2	13.13 (0.61)		
Trial 5	1	12.63 (1.12)	-0.331	0.750
	2	13.00 (0.71)		
Trials 1-5 Recall Total	1	55.00 (3.57)	-0.104	0.920
	2	55.13 (2.82)		
List B Free Recall Correct	1	8.25 (0.16)	0.000	1.000
	2	8.25 (0.92)		
Short-Delay Free Recall Correct	1	11.88 (1.26)	0.000	1.000
	2	11.88 (0.87)		
Long-Delay Free Recall Correct	1	12.29 (1.02)	0.679	0.522
	2	11.71 (0.97)		
Free-Recall Intrusions (Immediate & Delayed)	1	0.25 (0.16)	0.000	1.000
	2	0.25 (0.25)		
Total Repetitions (All Recall Trials)	1	1.63 (0.82)	-0.921	0.388
	2	2.63 (0.71)		
Repetition Hits	1	13.57 (0.68)	-0.367	0.726
	2	13.86 (0.74)		
Repetition False Positives	1	0.43 (0.30)	1.549	0.172
	2	0.14 (0.14)		

Table S2. CDC-94 criteria for ME/CFS [20].

**Inclusion criteria**
Clinically evaluated, medically unexplained fatigue of at least 6 months' duration that is:
– of new onset
– not a result of ongoing exertion
– not substantially alleviated by rest
A substantial reduction in previous levels of activity (occupational, educational, social, or personal)
The occurrence of 4 or more of the following symptoms:
– subjective memory impairment
– tender lymph nodes
– muscle pain
– joint pain
– headache
– unrefreshing sleep
– postexertional malaise (greater than 24 hours)
**Exclusion criteria**
– active, unresolved, or suspected disease likely to cause fatigue
– psychotic, melancholic, or bipolar depression (but not uncomplicated major depression)
– psychotic disorders
– dementia
– anorexia or bulimia nervosa
– alcohol or other substance misuse
– severe obesity
